# Supplementary material for: Towards Predictions of Interaction Dynamics between Cereal Aphids and Their Natural Enemies: A Review
Source: Insects. 2022 May 20;13(5):479. doi: 10.3390/insects13050479 (PMC9146300; doi:10.3390/insects13050479)
Supplement: Supplementary file 1 [file insects-13-00479-s001.zip › insects-1664752-supplementary.pdf]

Review

Towards Predictions of Interaction Dynamics between Cereal Aphids and Their Natural Enemies: A Review.

Eric Stell [1,2,] *, Helmut Meiss[2], Françoise Lasserre Joulin[2], Olivier Therond[1]

[1] LAE, Université de Lorraine, INRAE, F-68000 Colmar, France; olivier.therond@inrae.fr (O.T.)
[2] LAE, Université de Lorraine, INRAE, F-54000 Nancy, France; helmut.meiss@univ-lorraine.fr (H.M.); francoise.lasserre@univ-lorraine.fr (F.L.J.)
* Correspondence: eric.stell@univ-lorraine.fr

Supplementary Materials

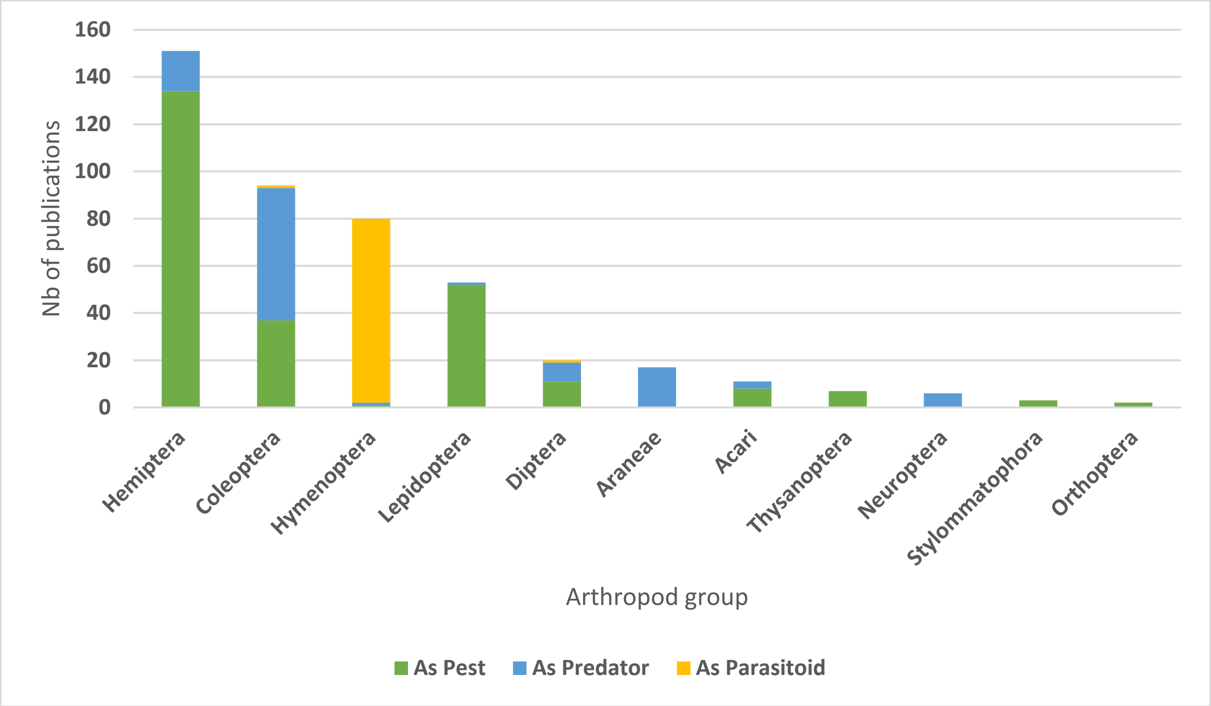

Figure S1. Number of publications for each type of ecological role after the second selection (343 **publications**). Hemiptera are the most studied pest, which motivated our choice to focus our review on aphids.

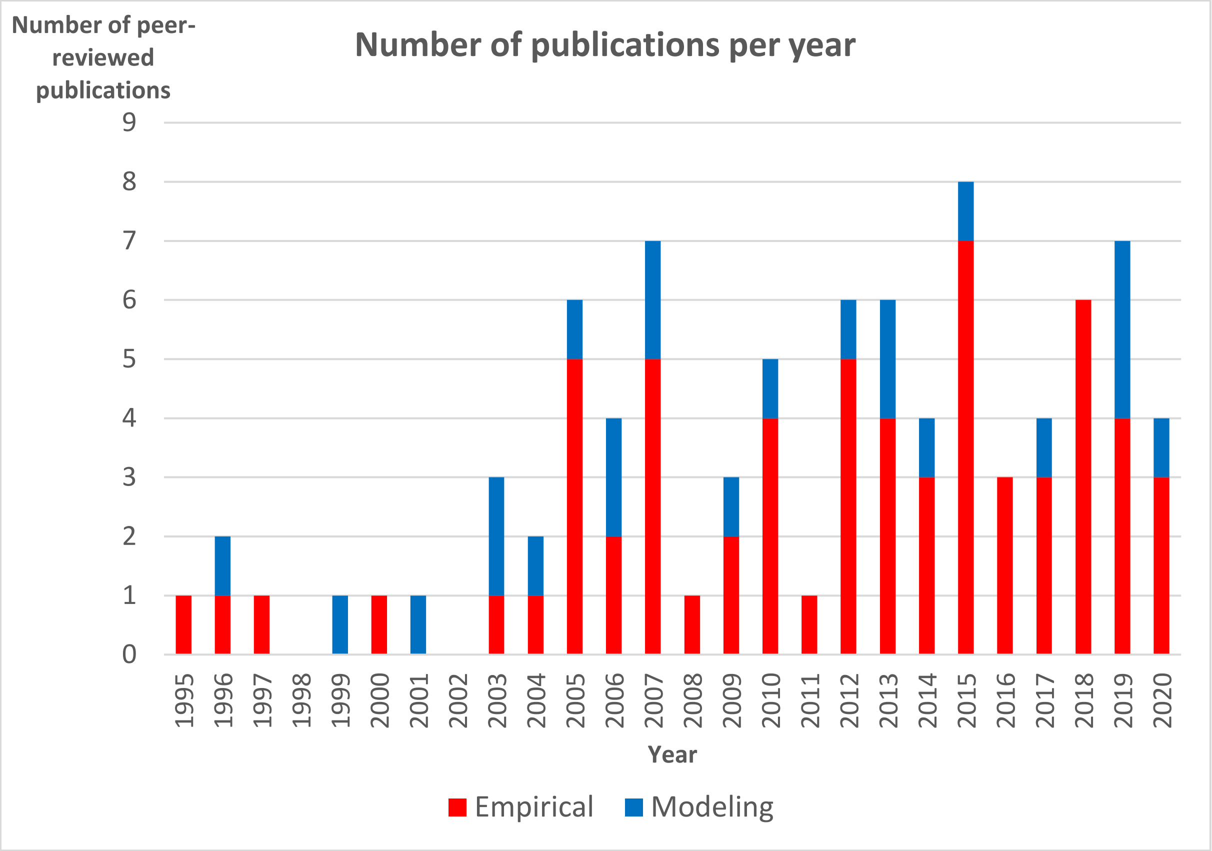

Figure S2. Number of peer-reviewed empirical and modeling publications per year that studied the dynamics of aphids, their natural enemies, and/or their interactions per year.

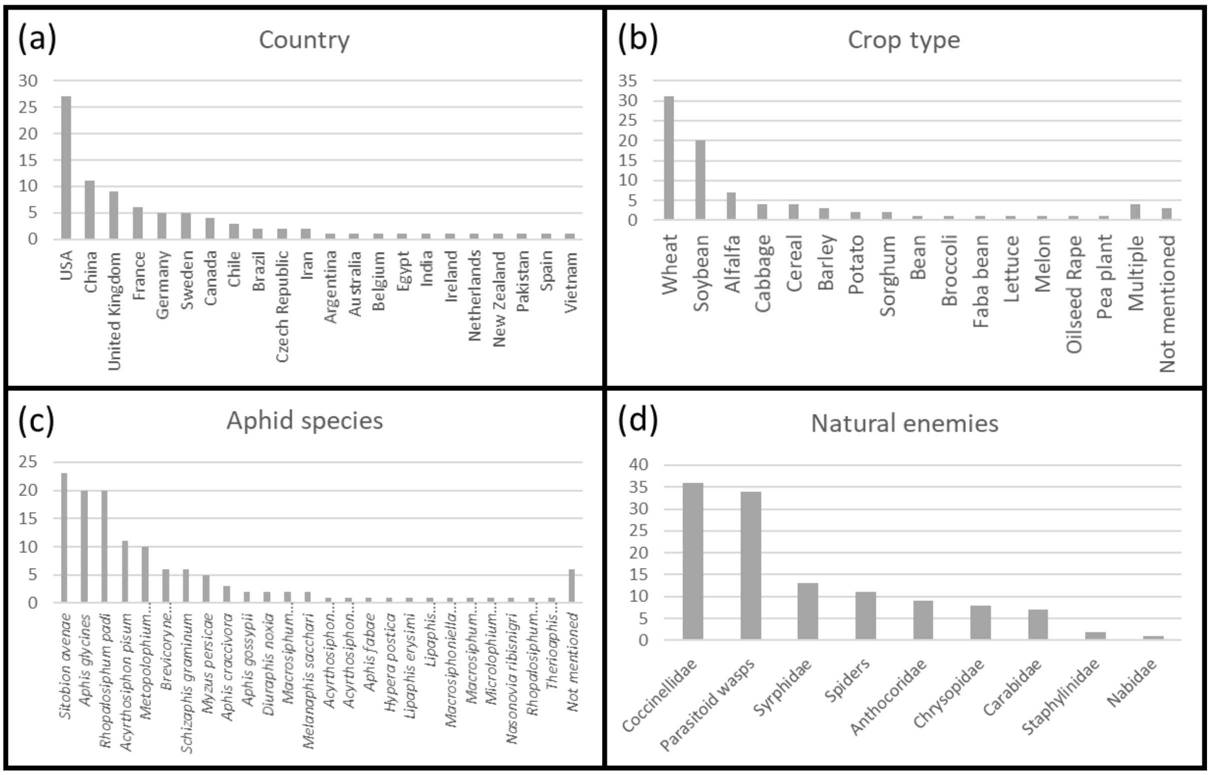

Figure S3. Characteristics of the 87 selected publications analyzing the dynamics of aphids, their natural enemies, and/or their interactions: (a) number of publications per country; (b) crop types studied; (c) aphid species studied; (d) natural enemy groups studied.

Table S1. Measurements and references for predictor indicators of aphid abundance.

		Response variable															
		Predation					Parasitism					Migration and flux					TOTAL
	Predictor variable	Pos		NS	Neg		Pos		NS	Neg		Pos		NS	Neg		
Category		Emp	Mod	Emp	Emp	Mod	Emp	Mod	Emp	Emp	Mod	Emp	Mod	Emp	Emp	Mod	
Climate	Temperature					1	1										2
Farming system type	Agroforestry				1										1		2
	% intercropping						1										1
	Agricultural intensification									1							1
	Crop type													1			1
	% natural borders						1										1
Insect	Aphid abundance	2				1		1	1			1		1			7
	Enemy alternative resources				1		1	1									3
	Enemy abundance											1			1		2
	Enemy arrival time					1											1
	Aphid competition												1				1
	Aphid growth rate												1				1
Landscape structure	Landscape complexity	1					1			1							3
	Landscape heterogeneity						1										1
	% grassland														1		1
	% maize											1					1
	Semi-natural habitat proximity			1													1
Period or Season	Timing in season	1			1		1								1		4
Plant phenology	Plant stage											1					1
	TOTAL =			11					12					12			35

Table S2. Ranking of predictor indicators in publications studying the effects of multiple predictors on aphid abundance. In each row, the predictors are ranked from level 1, the most important, to nonsignificant, the least important predictor in the study. We then tried to regroup publications studying the same predictor indicators (here as a to j).

	Ranking				
Publication	1	2	3	Nonsignificant	group
Alhmedi et al. (2009)	%SNH	Crop_type			a
Costamagna et al. (2011)	NE abundance	Aphids abundance			b
Costamagna et al. (2015)	Predation	%SNH	%grassland		c
Dahlin et al. (2018)	Crop_type			NE abundance	d
Fidelis et al. (2019)	Humidity; plant stage	Precipitation; Temperature	NE abundance		e
Kataria et al. (2017)	Humidity	Temperature	Precipitation		e
Merrill et al. (2012)	Crop_type	Precipitation			f
Roschewitz et al. (2005)	Agricultural Intensification			Landscape complexity	g
Seiter et al. (2019)	Insecticide Use	Planting date			h
Whitney et al. (2016)	Precipitation	%SNH			i
Zhao et al. (2019)	%SNH	Landscape complexity			j

Table S3. Ranking of predictor indicators in publications studying the effects of multiple predictors on aphids' natural enemies' abundances. In each row, the predictors are ranked from level 1, the most important, to nonsignificant, the least important predictor in the study. We then tried to regroup publications studying the same predictor indicators (here as a to k).

	Ranking				
Publication	1	2	3	Nonsignificant	group
Bianchi et al. (2007)	Fertilizer use	%SNH; Aphids abundance			a
Chaplin-Kramer et al. (2012)	Landscape complexity			Landscape complexity (syrphidae)	b
Raymond et al. (2015)	Landscape complexity (Coccinellidae)			Landscape complexity (Carabidae)	b
Elliott et al. (2000)	Aphids abundance (Coccillidae, larvae)	Aphids abundance (Coccillidae, adults)		Aphids abundance (NE)	c
Hesler et al. (2014)	Aphids abundance (Coccillidae)	Aphids abundance (NE in plants)		Aphids abundance (O. insidosus)	c
Rhainds et al. (2010)	Aphids abundance (Coccillidae)			Aphids abundance (Orius)	c
Evans et al. (2007)	Aphids abundance			NE Alternative Resources	d
Yoo et al. (2009)	NE Alternative Resources (Orius)			Aphids abundance (Orius)	d
Fidelis et al. (2019)	Humidity (Coccinellidae)	Aphids abundance (Syrphidae)	Humidity (spiders)		e
Gagic et al. (2012)	Agriculture Intensification (A. rhopalosiphi)	Agriculture intensification (E. plagiator)			f
Ghahramani et al. (2019)	Mechanical Practices (Coccinellidae)			Mechanical Practices (Carabidae)	g
Jacometti et al. (2010)	NE Alternative Resources (All NE)			NE Alternative Resources (Parasitoids)	h
Kataria et al. (2017)	Humidity (Coccinellidae)	Precipitation (Coccinellidae)	Temperature (Coccinellidae)		i
Lundgren et al. (2013)	Insecticide use (foliar-dwelling predators)			Insecticide use (soil surface dwelling predators)	j
Zhao et al. (2019)	%SNH	Landscape complexity			k

Table S4. Ranking of predictor indicators in publications studying the effects of multiple predictors on aphid population growth rate. In each row, the predictors are ranked from level 1, the most important, to nonsignificant, the least important predictor in the study. We then tried to regroup publications studying the same predictor indicators (here as a to d).

	Rank			
Publication	1	2	Nonsignificant	group
Bommarco et al. (2007)	Aphids abundance		Precipitation; Temperature	a
Chen et al. (1997)	Predator abundance		Parasitoids abundance; aphids abundance; plant stage; temperature; precipitation	a,c
Costamagna et al. (2006)	Predator abundance		Agricultural intensification	b
Elliott et al. (2000)	Predator abundance		Aphids abundance	c
Raymond et al. (2015)	Time in season	Predator abundance		d

Table S5. Studies testing ecological hypotheses concerning the effects of abiotic factors, agricultural practices, landscape, and biodiversity descriptors. These hypotheses were directly enunciated in the publications or deduced from their results and/or discussions.

Hypothesis theme	Ecological Hypothesis	Hypothesis tested in empirical studies			Hypothesis included in a modeling study
		Validated	Not validated	Variable conclusions	
Agricultural system	Intercropping influence on the dynamic of pest–natural enemy interactions	Amini et al. (2012), Arshad et al. (2018), Dahlin et al. (2018), Liu et al. (2017), Lundgren et al. (2013), Zhou, Chen, Chen et al. (2013), Zhou, Chen, Liu et al. (2013) (*n* = 7)			
	Farming system influence on the dynamic of pest–natural enemy interactions (Org vs. Conv; Fertilizer use; crop rotations; insecticides/plant extract)	Ali et al. (2018), Gagic et al. (2012), Rusch et al. (2013), Tran et al. (2016), Wang et al. (2015), Zumoffen et al. (2012) (*n* = 6)	Costamagna et al. (2006), Roschewitz et al. (2005), Rusch et al. (2013) (*n* = 3)		
Land use, landscape effect	Proximity between fields and semi-natural habitats; enhanced biocontrol; easier natural enemy dispersal; natural enemy reservoir	Ahlmedi et al. (2009), Arshad et al. (2018), Costamagna et al. (2015) (*n* = 3)			Bianchi et al. (2007), Bianchi et al. (2003) (*n* = 2)
	Aphid colonization rate correlated to abundance and distance to aphid source (forests, some crops…)	Gilabert et al. (2017), Jonsson et al. (2016), Whitney et al. (2016) (*n* = 3)			
	Predators are more effective earlier in the season in complex landscapes	Raymond et al. (2015) (*n* = 1)	Roschewitz et al. (2005) (*n* = 1)		
	Increasing landscape heterogeneities increase the stability of biotic interactions	Zhao et al. (2015) (*n* = 1)			
Aphids-only	Initial aphid density in the early season helps to predict aphid population throughout the season	Donaldson et al. (2007), Jonsson et al. (2016), Rhainds et al. (2010), Bommarco et al. (2007) (*n* = 4)		Costamagna et al. (2011) (*n* = 1)	

	Aphid spatial dispersion is driven by intrinsic reproductive rate				Paulson et al. (2009) (*n* = 1)
	Pest population dynamics are characterized by catastrophe behavior				Piyaratne et al. (2013) ,Wu et al. (2014) (*n* = 2)
	Aphid peak population occurs without seasonality	Fidelis et al. (2019) (*n* = 1)			
Natural enemies' impact on aphids	Enemy density is a major factor influencing aphid dynamics	Ali et al. (2018), Chen et al. (1997), Donaldson et al. (2007), Hesler et al. (2014), Karley et al. (2003), Miao et al. (2007), Amini et al. (2012), Costamagna et al. (2011), Gross et al. (2005), Winder et al. (2005) (*n* = 10)			Bahlai et al. (2013), Cursdotter et al. (2019) (*n* = 2)
	Predator effectiveness depends on initial aphid population	Rutledge et al. (2005) (*n* = 1)			
Timing of regulation	Earlier establishment of natural enemies in crops enhances biocontrol	Ali et al. (2018), Bortolotto et al. (2015), Costamagna et al. (2006), Costamagna et al. (2015), Fox et al. (2005), Leblanc et al. (2018), Raymond et al. (2015), Rhainds et al. (2010), Tenhumberg et al. (1995), Yoo et al. (2009) (*n* = 10)		Rutledge et al. (2005) (*n* = 1)	Gebauer et al. (2015), Miksanek and Heimpel (2019), Ro and Long (1999) (*n* = 3)
	Generalist natural enemies are more efficient in the early season when specialists are scarce	Fox et al. (2005), Ortiz-Martinez et al. (2020) (*n* = 2)	Khodeir et al. (2020) (*n* = 1)		
	There is a synchrony between aphids' population peak and predators' voracity peak	Tenhumberg et al. (1995), Rhainds et al. (2010) (*n* = 2)			

Spatial association between aphids and natural enemies	Natural enemies respond spatially to aphid population density in field	Donaldson et al. (2007), Elliott et al. (2000), Evans et al. (2007), Fidelis et al. (2019), Holland et al. (2004), Liu et al. (2017) Perez-Rodriguez et al. (2015), Winder et al. (2005) (*n* = 8)	Chen et al. (1997), Holland et al. (2004), Gross et al. (2005) (*n* = 3)		
Resource abundance	Alternative resources (flowers, prey) positively impact natural enemies' populations early in the season	Desneux et al. (2008), Evans et al. (2007), Jacometti et al. (2010) (n = 3)	Evans et al. (2007) (*n* = 1)	Desneux et al. (2008) (*n* = 1)	Vollhardt et al. (2010) (*n* = 1)
Plant phenology	Aphid population dynamics are linked to plant phenology throughout the cultural season (plant growth, plant quality)	Chen et al. (1997), Costamagna et al. (2007), Karley et al. (2003), Seiter et al. (2019) (*n* = 4)			Bahlai et al. (2013), Newman et al. (2003), Newman (2005), Wang et al. (2019) (*n* = 4)
Weather	Temperature is a main driver of pest–natural enemy interactions (by changing intrinsic species parameters and synchrony of interactions)	Bortolotto et al. (2015), Meisner et al. (2014), Whitney et al. (2016), Wang et al. (2015) (*n* = 4)	Bommarco et al. (2007), Perez-Rodriguez et al. (2015) (*n* = 2)		Bianchi et al. (2003), Cursdotter et al. (2019), Gebauer et al. (2015), Newman (2004), Newman (2005), Newman (2006), Merrill and Peairs (2012), Plantegenest et al. (1996), Preedy et al. (2020), Wu et al. (2014) (*n* = 10)
